# Depression, anxiety, and stress in breast cancer patients: prevalence, associated risk factors, and clinical correlates

**DOI:** 10.1038/s41598-025-17285-7

**Published:** 2025-08-24

**Authors:** Amirhossein Hessami, Erfan Ghadirzadeh, Shayan Ashrafi, Farima Taghavi, Forouzan Elyasi, Mobina Gheibi, Ehsan Zaboli, Reza Alizadeh-Navaei, Fatemeh Akbari Gelvardi, Akbar Hedayatizadeh-Omran, Mahmood Moosazadeh

**Affiliations:** 1 Non-communicable Disease Institute, Mazandaran University of Medical Sciences, Sari, Iran; 2 Student Research Committee, School of Medicine, Mazandaran University of Medical Sciences, Sari, Iran; 3 School of Medicine, Shiraz University of Medical Sciences, Shiraz, Iran; 4 Sexual and Reproductive Health Research Center, Psychiatry and Behavioral Sciences Research Center, Addiction Institute, Mazandaran University of Medical Sciences, Sari, Iran; 5 Student Research Committee, School of Allied Medical Sciences, Mazandaran University of Medical Sciences, Sari, Iran; 6 Gastrointestinal Cancer Research Center, Non-communicable diseases institute, Mazandaran University of Medical Sciences, Sari, Iran; 7 PhD in Psychology, Vice Chancellor for Research and Technology, Mazandaran University of Medical Sciences, Sari, Iran; 8 Psychosomatic Research Center, Faculty of Medicine, Imam Khomeini Hospital, Mazandaran University of Medical Sciences, Sari, Iran

**Keywords:** Breast neoplasms, Depression, Anxiety, Psychological stress, Cancer, Diseases, Health care, Medical research, Oncology, Psychology, Psychology

## Abstract

Overall, the prevalence and determinants of various forms of psychological distress such as depression and anxiety in breast cancer (BC) patients exhibit conflicting results, with some factors underexplored. The present study aimed to assess the prevalence and determinants of depression, anxiety, and stress among BC patients. This cross-sectional study assessed depression, anxiety, and stress in BC patients in Mazandaran, Iran, using the DASS-21 questionnaire, which showed high reliability (Cronbach’s alpha: 0.83–0.90). Data were collected via phone interviews and web-based surveys, and analyzed using SPSS software version 27. Demographic, clinical, and socioeconomic determinants were evaluated via logistic regression. This study included 283 BC patients, predominantly aged 40–44 (34.3%), married (95.4%), unemployed (73.9%), and urban residents (76.7%). Most patients received chemotherapy (94.3%), with 30.4% at Stage 1 and 43.1% Grade 2 tumors. Depression, anxiety, and stress prevalence were high (46.6%, 56.9%, and 51.9%, respectively). Key determinants included the absence of second-degree BC family history (linked to higher depression and stress, ORs: 1.86 and 2.15), urban living (higher depression and stress, ORs: 3.06 and 2.19), inadequate income (higher depression and stress, ORs: 2.62 and 1.94), and mastectomy (higher depression and stress, ORs: 3.28 and 1.90) versus breast-conserving surgery. The high prevalence of psychological distress in BC patients, particularly among those without second-degree family history, urban residents, individuals with inadequate income, and mastectomy recipients, underscores the critical need for routine psychological screening and early intervention in these high-risk groups.

## Introduction

Breast cancer (BC) represents a profound challenge not only to physical health but also to mental well-being, precipitating conditions such as depression and anxiety among patients. These psychological burdens can significantly affect the quality of life, treatment adherence, and overall prognosis, making it imperative to understand their prevalence and the factors that contribute to the odds of their occurrence^1,2^. However, research on the prevalence and determinants of depression and anxiety in BC patients reveals a landscape marked by substantial inconsistency.

Psychological distress in BC patients arises from the interplay of biological, psychological, and social factors^3,4^. Biological factors, including tumor characteristics (e.g., stage and grade) and treatments such as surgery type or chemotherapy, can induce physical symptoms like pain or fatigue that contribute to heightened depression, anxiety, and stress through neuroendocrine and immune pathways^5^. Psychological factors encompass individual coping mechanisms, cognitive appraisals, and resilience; for example, younger age or absence of family history may amplify distress by increasing perceived threat or reducing preparedness for the diagnosis^6^. Social factors involve socioeconomic elements (e.g., income adequacy, employment, marital status) and environmental contexts (e.g., urban vs. rural residence), which modulate access to support networks and resources, thereby influencing emotional adjustment and vulnerability to distress. These interactions provide a comprehensive rationale for examining these determinants, addressing underexplored inconsistencies in prior research.

The prevalence of depression and anxiety among BC patients varies dramatically across studies, reflecting differences in methodology, population characteristics, and assessment timing. While Álvarez-Pardo et al.^7^ reported a high prevalence of anxiety in 94.44% and depression in 69.18% of Mexican patients using the Hospital Anxiety and Depression Scale (HADS), Tsaras et al.^8^ and Benallel et al.^9^ found substantially lower rates (38.2% and 26% depression, and 32.2% anxiety, based on Patient Health Questionnaire-2 (PHQ-2), Mini International Neuropsychiatric Interview (MINI) test, and Generalized Anxiety Disorder-2 (GAD-2).

The factors influencing depression and anxiety in BC patients, determinants such as age, marital status, employment, family history, and surgery type, also exhibit significant inconsistencies across studies, reflecting the multifaceted nature of psychological distress.

Younger age was associated with distress in some studies^9–11^while others reported higher risk in older patients or no association^7,8^. While single status correlated with anxiety in Mexican patients^7^marital quality (rather than status) was determinative in Chinese patients^12^and no association was found in Moroccan and Greek population^8,9^. Employment showed paradoxical associations (unemployment was protective in Mexico but a risk factor in China) or no associations^7,9,12^while income adequacy predicted distress where measured^13^. Family history of BC shows a weak influence, with one study suggesting a slight benefit for depression, while other studies found no correlation^7^. Surgery type also yields mixed results with mastectomy (compared to breast-conservative surgery) linked to higher anxiety in Breidenbach et al.’s study but not in Álvarez-Pardo et al.’s study^7,10^.

In Iran, BC represents the most prevalent malignancy among women, with psychological distress rates substantially exceeding global averages; for instance, meta-analyses indicate depression prevalence at 66% and anxiety at 53.2% among Iranian BC patients, compared to lower rates in Western or other Asian cohorts^14^. Socio-cultural factors unique to Iran, including pervasive mental health stigma, limited utilization of psychological services (with only 28% of distressed BC survivors accessing care despite 70% perceiving a need), and reliance on religious or familial coping mechanisms, may distinctly modulate risk factors and barriers, underscoring the necessity for localized studies to guide culturally attuned screening and interventions^14–16^.

Overall, the prevalence and determinants of various forms of psychological distress (such as depression, anxiety, and stress) in BC patients exhibit conflicting results, with some factors (e.g., family history) underexplored. Discrepancies may stem from cultural, methodological, or contextual differences, highlighting the need for more standardized research. Thus, the present study aimed to assess the prevalence and determinants of depression, anxiety, and stress among BC patients.

## Methods

### Study design and setting

This cross-sectional study was conducted in Mazandaran Province, located in northern Iran. To adhere to the standards of a cross-sectional study, the STROBE checklist was utilized to report the results of this study^17^.

### Study population

The study population comprised all women diagnosed with BC in Mazandaran, Iran. Following approval of the ethical committee (Ethical code: IR.MAZUMS.REC.1402.621), an Excel-formatted list of women diagnosed with BC between March 2021 and June 2024 in Mazandaran, Iran, was obtained from the Cancer Registry Center of Mazandaran University of Medical Sciences.

### Inclusion criteria

The inclusion criteria were as follows:

- BC patients aged between 30 and 59 years.
- Completion of their treatment plans at the time of completing the questionnaire.
- Pathologically confirmed diagnosis of BC.
- Literacy sufficient to read texts and the ability to use the internet, mobile devices, or personal computers.

### Exclusion criteria

The exclusion criteria included:

- BC patients undergoing active treatment at the time of the study.
- Inability to communicate effectively.
- Literacy insufficient to read texts and the inability to use the internet, mobile devices, or personal computers.
- Lack of valid contact information (phone number or address).
- Unwillingness to participate in the study.
- Incomplete questionnaire responses.

### Sample size estimation

The sample size was determined based on the findings of Musarezaie et al.^18^which reported a depression prevalence of 34.5% among BC patients in Isfahan, Iran. Considering this estimate, with a 95% confidence level (CI) and a precision of 0.06, the minimum required sample size was calculated as 241 using the single proportion formula. Using a consecutive sampling approach, all eligible patients from the registry were contacted via telephone. Eligibility was assessed through phone screening based on inclusion/exclusion criteria. Enrollment continued until the sample size target was met. Ultimately, considering 10% attrition rate, 283 eligible participants completed the questionnaire.

### Data collection period

Questionnaires were administered from February 4, 2024, to April 19, 2024.

### Questionnaire

The Depression, Anxiety, and Stress Scale − 21 (DASS-21) was used in the present study, which is a self-reporting instrument developed to assess emotional distress across three domains^19^: Depression, Anxiety, and Stress. The DASS-21 is a shortened version of the DASS-42, comprising 21 items (seven per subscale), and employs a four-point Likert-type response format. The DASS-21 demonstrates high internal consistency and discriminant validity, making it a reliable instrument for both research and clinical applications^20^. The Persian-version of DASS-21 has demonstrated acceptable internal consistency, with Cronbach’s alpha values of 0.79 for anxiety, 0.91 for stress, and 0.93 for depression, and satisfactory test-retest reliability across all three subscales (ranging from 0.740 to 0.881) both in previous studies in Iran and in the present study (with Cronbach’s alpha values of 0.83 for anxiety, 0.88 for stress, and 0.90 for depression).

### Data collection process

A trained clinical psychologist conducted the data collection process. The DASS-21 questionnaire was designed in a web-based format. The interviewer contacted potential participants via telephone, using identification details recorded in the Excel file. The purpose of the study was explained to them, and participants were assured of confidentiality, privacy, and adherence to ethical research principles. They were also reminded that their participation was entirely voluntary.

Participants were informed that they were not required to make an immediate decision regarding their involvement and were encouraged to seek clarification from the research team, relatives, friends, or any other trusted individuals before consenting. Additionally, they were instructed to carefully review the informed consent form, which was prepared in accordance with Helsinki Declaration guidelines, and to indicate their agreement by selecting the “Yes” option in the web-based format if they wished to proceed.

The interviewer then provided a detailed explanation of the questionnaire to ensure that participants fully understood its contents. Following this, the web-based survey was distributed to eligible candidates. To address any potential queries, such as those related to completion procedures, response submissions, or technical issues, the research team implemented a follow-up protocol. Eligible participants independently completed the web-based DASS-21 survey after the initial explanatory phone call. No questionnaire items were administered or recorded during telephone contacts.

### Other demographic and clinical variables

Other demographically and clinically relevant variables such as age, marital status and age at marriage, residential place, first and second-degree family history of BC, occupation, educational level, number of pregnancies and age at first pregnancy, surgical type (mastectomy or breast-conservative), chemotherapy, stage, and grade of tumor were obtained from the Cancer Registry Center of Mazandaran University of Medical Sciences or patients health records and documents. Additionally, the participants were asked a question (“Is your level of financial income sufficient to cover the needs of yourself and your family? Yes or No”) during the web-based survey to assess their perspective on the efficiency of their income level.

### Statistics

The data were extracted from the web-based questionnaire and transferred to SPSS software version 27 for statistical analysis after data cleaning. Variables are described using number, frequency, mean, standard deviation, median, interquartile range (IQR), and minimum and maximum values. The internal consistency of the questionnaire was determined using Cronbach’s alpha. The frequency of depression, anxiety, and stress severity was presented based on the Likert scale. To estimate the prevalence of depression, anxiety, and stress, normal individuals were categorized into the “without disorder” group, while those with mild to very severe symptoms were classified into the “with disorder” group. Comparisons between individuals with and without depression, anxiety, and stress disorders, as well as grouped variables, were conducted using the Chi-square test or Fisher’s exact test. Also, factors associated with depression, anxiety, and stress disorders in women with BC were analyzed using Univariate and multivariate logistic regression. Additionally, variables with a significance level of less than 0.250 in the Univariate analysis were included in the multiple logistic regression model.

## Results

### Patient characteristics

A total of 283 patients with BC participated in the present study. The majority of participants were aged 40–44 years old (34.3%), married (95.4%) with age at first marriage of 18–22 years old (36.7%), had two pregnancies up to date (41.3%) with age at first pregnancy of 20–24 years old (26.9%), did not have a job (73.9%), resided in a city (76.7%), and had an educational level higher than diploma (47.0%) (Table 1). Approximately 18% and 30% of participants had a family history of BC in their first-degree and second-degree relatives, respectively. Additionally, 94.3% of participants had received chemotherapy, and 65% had undergone breast-conserving surgery. The majority of BC patients were of Stage 1 (30.4%) and Grade 2 tumors (43.1%) (Table 2).

**Table 1:** Demographical baseline characteristics of participants in total, and based on depression, anxiety, or stress diagnoses. Data is presented as number (percent).

Variables		Total	Depression		P-value	Anxiety		P-value	Stress		P-value
			Yes (n: 132)	No (n: 151)		Yes (n: 161)	No (n: 122)		Yes (n: 147)	No (n: 136)	
Age	< 40	65 (23.0)	32 (49.2)	33 (50.8)	0.868	36 (55.4)	29 (44.6)	0.574	34 (52.3)	31 (47.7)	0.996
	40–44	97 (34.3)	42 (43.3)	55 (56.7)		60 (61.9)	37 (38.1)		50 (51.5)	47 (48.5)	
	45–49	76 (26.9)	36 (47.4)	40 (52.6)		39 (51.3)	37 (48.7)		39 (51.3)	37 (48.7)	
	≥ 50	45 (15.9)	22 (48.9)	23 (51.1)		26 (57.8)	19 (42.2)		24 (53.3)	21 (46.7)	
Marriage	Married	270 (95.4)	123 (45.6)	147 (54.4)	0.153	153 (56.7)	117 (43.3)	0.783	140 (51.9)	130 (48.1)	1.000
	Single	13 (4.6)	9 (69.2)	4 (30.8)		8 (61.5)	5 (38.5)		7 (53.8)	6 (46.2)	
Residence	Rural	66 (23.3)	22 (33.3)	44 (66.7)	0.016	31 (47.0)	35 (53.0)	0.067	27 (40.9)	39 (59.1)	0.049
	Urban	217 (76.7)	110 (50.7)	107 (49.3)		130 (59.9)	87 (40.1)		120 (55.3)	97 (44.7)	
Job	Yes	74 (26.1)	34 (45.9)	40 (54.1)	0.893	45 (60.8)	29 (39.2)	0.495	38 (51.4)	36 (48.6)	1.000
	No	209 (73.9)	98 (46.9)	111 (53.1)		116 (55.5)	93 (44.5)		109 (52.2)	100 (47.8)	
Education	< Diploma	69 (24.4)	33 (47.8)	36 (52.2)	0.895	41 (59.4)	28 (40.6)	0.818	38 (55.1)	31 (44.9)	
	Diploma	81 (28.6)	39 (48.1)	42 (51.9)		44 (54.3)	37 (45.7)		46 (56.8)	35 (43.2)	
	> Diploma	133 (47.0)	60 (45.1)	73 (54.9)		76 (57.1)	57 (42.9)		63 (47.4)	70 (52.6)	0.332
Adequate income?	Yes	103 (36.4)	34 (33.0)	69 (67.0)	0.001	56 (54.4)	47 (45.6)	0.535	44 (42.7)	59 (57.3)	0.020
	No	180 (63.6)	98 (54.4)	82 (45.6)		105 (58.3)	75 (41.7)		103 (57.2)	77 (42.8)	
FH-1st	Yes	52 (18.4)	27 (51.9)	25 (48.1)	0.443	32 (61.5)	20 (38.5)	0.536	28 (53.8)	24 (46.2)	0.878
	No	231 (81.6)	105 (45.5)	126 (54.5)		129 (55.8)	102 (44.4)		119 (51.5)	112 (48.5)	
FH-2nd	Yes	86 (30.4)	35 (40.7)	51 (59.3)	0.197	45 (52.3)	41 (47.7)	0.361	36 (41.9)	50 (58.1)	0.028
	No	197 (69.6)	97 (49.2)	100 (50.8)		116 (58.9)	81 (41.1)		111 (56.3)	86 (43.7)	
Age at first marriage	Never	13 (4.6)	9 (69.2)	4 (30.8)	0.366	8 (61.5)	5 (38.5)	0.748	7 (53.8)	6 (46.2)	0.886
	< 18	66 (23.3)	30 (45.5)	36 (54.5)		38 (57.6)	28 (42.4)		35 (53.0)	31 (47.0)	
	18–22	104 (36.7)	43 (41.3)	61 (58.7)		60 (57.7)	44 (42.3)		50 (48.1)	54 (51.9)	
	23–27	62 (21.9)	31 (50.0)	31 (50.0)		31 (50.0)	31 (50.0)		35 (56.5)	27 (43.5)	
	> 27	38 (13.4)	19 (50.0)	19 (50.0)		24 (63.2)	14 (36.8)		20 (52.6)	18 (47.4)	
Pregnancy	Yes	246 (86.9)	112 (45.5)	134 (54.5)	0.379	142 (57.7)	104 (42.3)	0.481	130 (52.8)	116 (47.2)	0.483
	No	37 (13.1)	20 (54.1)	17 (45.9)		19 (51.4)	18 (48.6)		17 (45.9)	20 (54.1)	
Age at first pregnancy	Never	37 (13.1)	20 (54.1)	17 (45.9)	0.875	19 (51.4)	18 (48.6)	0.539	17 (45.9)	20 (54.1)	0.929
	< 20	60 (21.2)	26 (43.3)	34 (56.7)		30 (50.0)	30 (50.0)		31 (51.7)	29 (48.3)	
	20–24	76 (26.9)	34 (44.7)	42 (55.3)		47 (61.8)	29 (38.2)		39 (51.3)	37 (48.7)	
	25–29	56 (19.8)	26 (46.4)	30 (53.6)		35 (62.5)	21 (37.5)		31 (55.4)	25 (44.6)	
	≥ 30	54 (19.1)	26 (48.1)	28 (51.9)		30 (55.6)	24 (44.4)		29 (53.7)	25 (46.3)	
Number of pregnancies	0	37 (13.1)	20 (54.1)	17 (45.9)	0.595	19 (51.4)	18 (48.6)	0.612	17 (45.9)	20 (54.1)	0.537
	1	65 (23.0)	29 (44.6)	36 (55.4)		34 (52.3)	31 (47.7)		31 (47.7)	34 (52.3)	
	2	117 (41.3)	58 (49.6)	59 (50.4)		73 (62.4)	44 (37.6)		68 (58.1)	49 (41.9)	
	3	36 (12.7)	14 (38.9)	22 (61.1)		19 (52.8)	17 (47.2)		17 (47.2)	19 (52.8)	
	> 3	28 (9.9)	11 (39.3)	17 (60.7)		16 (57.1)	12 (42.9)		14 (50.0)	14 (50.0)	

FH Family History.

**Table 2:** Clinical baseline characteristics of participants in total, and based on depression, anxiety, or stress diagnoses. Data is presented as number (percent).

Variables		Total	Depression		*P*-value	Anxiety		*P*-value	Stress		*P*-value
			Yes (*n*: 132)	No (*n*: 151)		Yes (*n*: 161)	No (*n*: 122)		Yes (*n*: 147)	No (*n*: 136)	
Surgery	BC	184 (65.0)	70 (38.0)	114 (62.0)	< 0.001	101 (54.9)	83 (45.1)	0.380	87 (47.3)	97 (52.7)	0.035
	Ma	99 (35.0)	62 (62.6)	37 (37.4)		60 (60.6)	39 (39.4)		60 (60.6)	39 (39.4)	
Chemotherapy	Yes	267 (94.3)	126 (47.2)	141 (52.8)	0.607	152 (56.9)	115 (43.1)	1.000	140 (52.4)	127 (47.6)	0.609
	No	16 (5.7)	6 (37.5)	10 (62.5)		9 (56.3)	7 (43.8)		7 (43.8)	9 (56.3)	
Stage	1	86 (30.4)	41 (47.7)	45 (52.3)	0.804	57 (66.3)	29 (33.7)	0.176	46 (53.5)	40 (46.5)	0.352
	2	67 (23.7)	33 (49.3)	34 (50.7)		33 (49.3)	34 (50.7)		33 (49.3)	34 (50.7)	
	3	63 (22.3)	26 (41.3)	37 (58.7)		34 (54.0)	29 (46.0)		28 (44.4)	35 (55.6)	
	UnK	67 (23.7)	32 (47.8)	35 (52.2)		37 (55.2)	30 (44.8)		40 (59.7)	27 (40.3)	
Grade	1	40 (14.1)	18 (45.0)	22 (55.0)	0.805	25 (62.5)	15 (37.5)	0.587	20 (50.0)	20 (50.0)	0.839
	2	122 (43.1)	54 (44.3)	68 (55.7)		72 (59.0)	50 (41.0)		61 (50.0)	61 (50.0)	
	3	41 (14.5)	19 (46.3)	22 (53.7)		20 (48.8)	21 (51.2)		21 (51.2)	20 (48.8)	
	UnK	80 (28.3)	41 (51.2)	39 (48.8)		44 (55.0)	36 (45.0)		45 (56.3)	35 (43.8)	

BC: Breast-Conservative, Ma: Mastectomy, FH: Family History, UnK: Unknown.

### Prevalence of depression, anxiety, and stress in BC patients

Table 3 shows the mean and SD, median, IQR, and range of DASS-21 scores for each subcategory. The mean and SD of DASS-21 scores for depression, anxiety, and stress were 12.20 ± 11.78, 11.49 ± 10.09, and 17.57 ± 11.48, respectively. Additionally, Cronbach’s alpha indicates good reliability of the DASS-21 questionnaire among our participants (Table 3). The prevalence of depression was 46.6%, with 8.5%, 16.3%, 7.8%, and 14.1% cases with mild, moderate, severe, and very severe depression. The prevalence of anxiety was 56.9%, with 7.8%, 17.7%, 12.7%, and 18.7% cases with mild, moderate, severe, and very severe anxiety. The prevalence of stress was 51.9%, with 13.8%, 12.0%, 12.4%, and 13.8% of cases categorized as mild, moderate, severe, and very severe stress, respectively.

**Table 3:** Mean, SD, median, IQR, range, and cronbach’s alpha in DASS-21 questionnaire.

Outcome	Mean	SD	Median	IQR(Q1-Q3)	Range (Min-Max)	Alpha-Cronbach (Reliability)
Depression	12.20	11.78	8	2–20	0–42	0.902
anxiety	11.49	10.09	8	4–16	0–42	0.839
Stress	17.57	11.48	16	8–26	0–42	0.880

### Determinants of depression, anxiety, and stress in BC patients

**Fig. 1:**
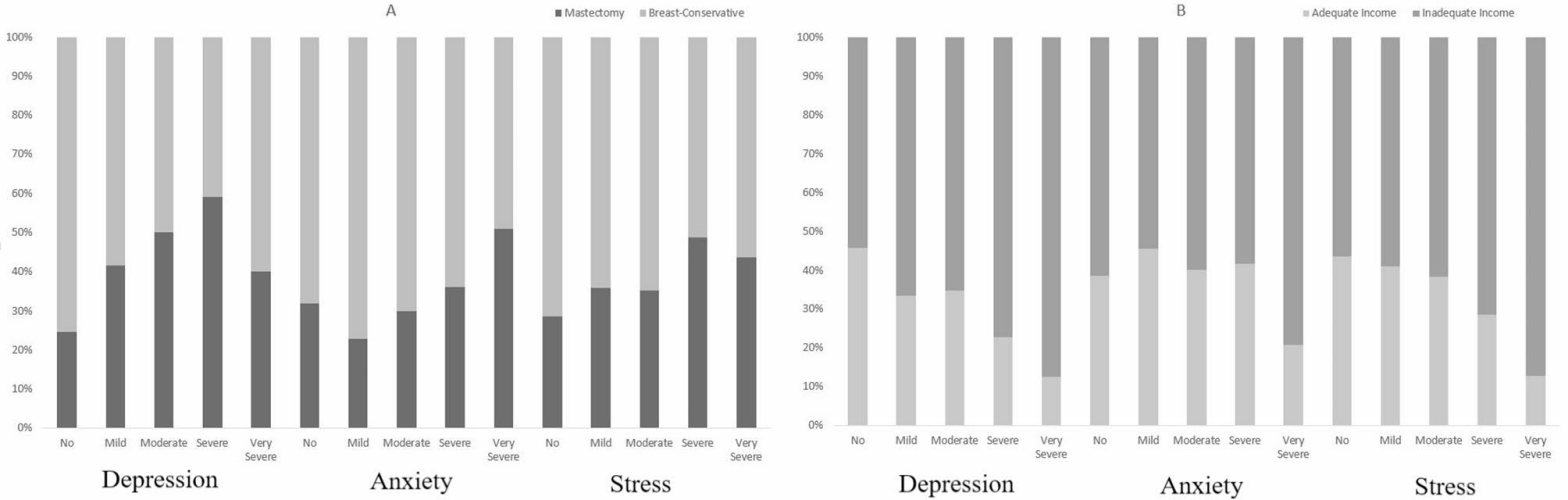
Distribution of BC patients based on severity of depression, anxiety, or stress in different types of surgery (**A**) and adequacy of income (**B**).

Table 4 demonstrates the results of Univariate and multivariate logistic regression. Our results indicated that participants without a family history of BC in their second-degree relatives had higher odds of depression (OR: 1.86, 95%CI: 1.06–3.27, P: 0.029) and stress (OR: 2.15, 95%CI: 1.25–3.68, P: 0.005). Also, participants living in urban areas have higher odds of depression (OR: 3.06, 95%CI: 1.61–5.83, P: 0.001) and stress (OR: 2.19, 95%CI: 1.21–3.96, P: 0.009); participants without adequate income had higher odds of depression (OR: 2.62, 95%CI: 1.52–4.53, P: 0.001), and stress (OR: 1.94, 95%CI: 1.16–3.24, P: 0.011) (Fig. 1); and participants who underwent mastectomy had higher odds of depression (OR: 3.28, 95%CI: 1.88–5.73, *P* < 0.001), and stress (OR: 1.90, 95%CI: 1.12–3.22, P: 0.017) compared to those who underwent breast-conservative surgery (Fig. 1).

**Table 4:** Logistic regression results on associating factors of depression, anxiety, and stress, in BC participants.

Variables		Univariate			Multivariate		
		OR	95% CI	*P*-value	OR	95% CI	*P*-value
Anxiety ^1^							
Stage	1	Ref					
	2	0.49	0.25–0.95	0.035	0.51	0.26–0.98	0.046
	3	0.59	0.30–1.16	0.129	0.62	0.31–1.22	0.168
	UnK	0.62	0.32–1.21	0.164	0.69	0.35–1.36	0.288
Residence	Rural	Ref					
	Urban	1.68	0.96–2.93	0.064	1.61	0.91–2.84	0.100
Depression ^2^							
FH-2nd	Yes	Ref					
	No	1.41	0.84–2.36	0.186	1.86	1.06–3.27	0.029
Residence	Rural	Ref					
	Urban	2.05	1.15–3.66	0.014	3.06	1.61–5.83	0.001
Adequate income?	Yes	Ref					
	No	2.42	1.46–4.01	0.001	2.62	1.52–4.53	0.001
Surgery type	BC	Ref					
	Ma	2.72	1.64–4.51	< 0.001	3.28	1.88–5.73	< 0.001
Marriage	Married	Ref					
	Single	2.68	0.80–8.94	0.107	3.47	0.92-13.00	0.064
Stress ^3^							
FH-2nd	Yes	Ref					
	No	1.79	1.07–2.99	0.026	2.15	1.25–3.68	0.005
Residence	Rural	Ref					
	Urban	1.78	1.02–3.12	0.042	2.19	1.21–3.96	0.009
Adequate income?	Yes	Ref					
	No	1.79	1.09–2.92	0.019	1.94	1.16–3.24	0.011
Surgery type	BC	Ref					
	Ma	1.71	1.04–2.81	0.033	1.90	1.12–3.22	0.017

^1^ (Nagelkerke R Square: 0.036, Hosmer-Lemeshow P-value: 0.951); ^2^ (Nagelkerke R Square: 0.198, Hosmer-Lemeshow P-value: 0.414); ^3^ (Nagelkerke R Square: 0.106, Hosmer-Lemeshow P-value: 0.993)

OR: Odds Ratio, CI: Confidence Interval, UnK: Unknown, BC: Breast-Conservative, Ma: Mastectomy, FH: Family History

## Discussion

The present study aimed to assess the prevalence and determinants of depression, anxiety, and stress among 283 BC patients. Using the DASS-21 scale, we found a prevalence of depression at 46.6%, with 8.5% mild, 16.3% moderate, 7.8% severe, and 14.1% very severe cases. Anxiety prevalence was 56.9%, with 7.8% mild, 17.7% moderate, 12.7% severe, and 18.7% very severe cases. Stress prevalence was 51.9%, with 13.8% classified as mild, 12.0% as moderate, 12.4% as severe, and 13.8% as very severe.

Key determinants included lack of family history of BC in second-degree relatives (depression OR: 1.86, 95% CI: 1.06–3.27; stress OR: 2.15, 95% CI: 1.25–3.68), urban living (depression OR: 3.06, 95% CI: 1.61–5.83; stress OR: 2.19, 95% CI: 1.21–3.96), inadequate perceived income (depression OR: 2.62, 95% CI: 1.52–4.53; stress OR: 1.94, 95% CI: 1.16–3.24), and mastectomy (depression OR: 3.28, 95% CI: 1.88–5.73; stress OR: 1.90, 95% CI: 1.12–3.22).

BC survivors frequently encounter depression, stress, and anxiety, which profoundly influence their quality of life. Understanding the mechanisms driving these mental health conditions is critical for devising effective interventions. Biological mechanisms significantly contribute to these mental health challenges. Cancer and its treatments often provoke systemic inflammation, a process implicated in depression. Research has demonstrated that elevated levels of inflammatory cytokines, such as interleukin-6 and c-reactive protein, correlate with increased depressive symptoms in BC patients^21,22^. Neurological effects also play a role; cognitive impairments, commonly termed “chemo brain,” arise from chemotherapy and may be associated with heightened stress and anxiety due to their impact on daily functioning^23^.

Biologically, inflammation underlies behavioral symptoms, with nuclear factor kappa B (NF-κB) and other mediators associating with depressive symptoms post-treatment^24^. Distinct inflammatory processes contribute to the symptom cluster of depression, fatigue, and sleep disturbances in survivors^25^. Additionally, covariation with the hypothalamic-pituitary-adrenal (HPA) axis and sympathetic nervous system exacerbates pain-depression-fatigue clusters in advanced cases^26^. At the molecular level, depression and BC share genetic bases and pleiotropic loci, suggesting causal links^27^. The IL-17/NF-κB pathway also drives BC-related depression^28^. These biological changes highlight the physiological impact of BC and its management on mental well-being.

Surgical interventions, such as mastectomy, often lead to body image disturbances, which may be linked to depression and reduced self-esteem^29^. Social mechanisms additionally shape the mental health landscape for survivors. Social support serves as a protective factor, with evidence suggesting that patients with less support networks experience higher levels of depression and anxiety^30^. Conversely, the financial burden imposed by treatment costs can intensify depression and anxiety, particularly among those in lower socioeconomic brackets^31^.

The interplay of these mechanisms reveals a complex etiology for depression, stress, and anxiety after BC. Biologically, inflammation and treatment-related changes disrupt physiological homeostasis, while psychologically, the trauma of diagnosis and its aftermath challenge emotional resilience. Socially, support systems and societal pressures either mitigate or exacerbate these effects. This multifaceted nature necessitates a comprehensive approach to care, addressing both the physiological and psychosocial needs of survivors. To contextualize our findings, we compare them with six recent studies: Álvarez-Pardo et al.^7^ Tsaras et al.^8^ Benallel et al.^9^ Breidenbach et al.^10^ Soqia et al.^11^ and Guo et al.^12^. These studies vary in sample size, assessment tools, and cultural settings, providing a broad basis for comparison.

Our study’s prevalence rates are higher than some studies but lower than others. Álvarez-Pardo et al.^7^ reported 69.18% of 198 BC patients scoring ≥ 8 on the HADS-D (depression), with 10.60% classified as pathological, and 94.44% scoring ≥ 8 on HADS-A (anxiety), far exceeding our rates. Guo et al.^12^ found 63.6% depression, 60.2% anxiety, and 36.9% stress among 176 metastatic BC (MBC) patients using DASS-21, with depression and anxiety higher than ours, though stress was lower. In contrast, Tsaras et al.^8^ reported lower rates among 152 patients, 38.2% depression and 32.2% anxiety, using PHQ-2 and GAD-2. Benallel et al.^9^ found 26% depression in 100 patients via the MINI test, the lowest among these studies. Soqia et al.^11^ reported 35% depression and 35.6% anxiety in 500 patients using PHQ-2 and GAD-2, also lower than our findings. Breidenbach et al.^10^ noted that 34.9% of 164 patients had mild to severe depressive symptoms five to six years post-diagnosis, with anxiety scores indicating mild levels, suggesting a lower burden compared to our acute-phase data.

These discrepancies likely stem from the use of differing assessment tools. The DASS-21, used in our study and Guo et al.’s, captures a broad spectrum of symptom severity, potentially inflating prevalence compared to PHQ-2/GAD-2 (Tsaras et al. and Soqia et al.) or MINI (Benallel et al.), which are screening tools with specific cut-offs. HADS, used by Álvarez-Pardo et al., has a lower threshold (≥ 8) for detecting symptoms, explaining its higher rates. Cultural differences across Mexico, Greece, Morocco, Germany, Syria, and China may also influence reporting, with stigma or resilience varying by region, and with Iranian patients potentially internalizing distress.

Our study found no significant association between age and psychological distress. Tsaras et al.^8^ linked younger age to higher depression and anxiety risk, with rural residents aged 40–59 most affected. Benallel et al.^9^ reported age 20–40 as a depression risk factor, with no depression in those over 60. Breidenbach et al.^10^ found women under 50 with higher depression (Coef: 1.17) and anxiety (Coef: 1.08) in linear regression. Soqia et al.^11^ noted that younger age (≤ 45) increased depression (OR: 1.485, 95% CI: 1.012–2.179) and anxiety (OR: 1.646, 95% CI: 1.125–2.408). On the other hand, Guo et al.^12^ and Aggeli et al.^32^ found no age effect. Our lack of age association may reflect a narrow age range or cultural factors mitigating age-related distress in our predominantly middle-aged sample.

With 95.4% of our participants married, we found no association with marital status. Tsaras et al.^8^ linked marital status to depression and anxiety, though specifics were unclear. Soqia et al.^11^ found divorced (OR: 6.031, 95% CI: 1.1751–23.151) and widowed (OR: 3.300, 95% CI: 1.237–8.803) women with higher depression, and widowed with higher anxiety (OR: 2.742, 95% CI: 1.075–6.993). Guo et al.^12^ identified poor marriage quality as a predictor of depression, anxiety, and stress. Álvarez-Pardo et al.^7^ found single women with higher anxiety scores (12.54 vs. 11.57), while Vahdaninia et al.^33^ found being single as a protective factor against depression. Our high marriage rate may have masked variability, unlike studies with diverse marital statuses. This may reflect Iran’s exceptionally high marriage rate (95.4%) limiting variability, and cultural normalization of midlife health challenges in our 40-44yo dominant subgroup.

Education beyond high school (47.0%) was not a determinant in our study. Tsaras et al.^8^ associated lower education with higher depression and anxiety risk. Breidenbach et al.^10^ found that university education is protective against depression (Coef: -1.15). Other studies found no link^9,11,12,32^. Educational differences across populations or cultural valuation of education may explain these inconsistencies. Inadequate income increased depression (OR: 2.62) and stress (OR: 1.94) in our study, similar to Sebro et al.‘s findings, which revealed that lower-income BC patients were at higher risk for depression in Ethiopia^13^. Guo et al.^12^ also found income predictive of depression. Tsaras et al.^8^ and others did not report income effects, possibly due to differing socioeconomic contexts or healthcare access^32^as Benallel et al.‘s^9^ insured sample showed no income impact.

Notably, we found no family history of BC in second-degree relatives linked to higher depression (OR: 1.86) and stress (OR: 2.15); however, Álvarez-Pardo et al.^7^ reported no significant difference in these outcomes (depression: 8.61 vs. 8.01; anxiety: 12.12 vs. 11.68). Other studies reported no association. This may reflect greater shock or isolation in patients without familial BC experience, a factor less explored elsewhere. Contrary to assumptions about genetic risk buffering psychological impact, patients without second-degree family histories showed significantly higher depression. This suggests that familial cancer exposure may foster psychological preparedness, whereas those lacking this background experience greater diagnostic shock and isolation.

Mastectomy increased depression (OR: 3.28) and stress (OR: 1.90) compared to breast-conserving surgery in our study. Breidenbach et al.^10^ linked mastectomy to higher anxiety (Coef: 0.91). Álvarez-Pardo et al.^7^ and Cáceres et al.^34^ found no difference in scores (depression: 8.18 vs. 8.15, anxiety: 12.08 vs. 11.62 in Álvarez-Pardo et al.’s; and 10.29 ± 7.83 vs. 10.95 ± 8.09 Beck Depression Inventory score in Cáceres et al.’s). Body image concerns or counseling differences may drive our findings, contrasting with Álvarez-Pardo’s null result.

Psychosocially, mastectomy induces body image disturbances, feelings of incompleteness, and insecurity, heightening anxiety and depression^35,36^. Cognitive attitudes like helplessness/hopelessness, global stress perceptions, intrusive cancer-related thoughts, financial difficulties, and neuroticism amplify symptoms^37,38^. Lack of social support and poor coping exacerbate distress, though interventions like pre-surgical psychological support and breast reconstruction aid adaptation^39^.

Urban living increased depression (OR: 3.06) and stress (OR: 2.19) in our study, unlike Tsaras et al.^8^where rural residents had higher risks (depression OR: 2.6; anxiety OR: 3.8). Urban stressors (e.g., isolation, cost) versus rural healthcare access issues may explain this divergence. We propose this stems from urban Iran’s ‘double burden’: financial pressures from treatment costs in cities, compounded by reduced communal support in anonymized urban environments. This contrasts with rural settings where traditional support networks remain intact despite healthcare access challenges.

Employment was not a determinant in our study (73.9% jobless). Álvarez-Pardo et al.^7^ found unemployed women with lower depression scores (7.89 vs. 8.61), while Guo et al.^12^ found working status predictive of depression. Our high unemployment rate may limit the detection of variability.

Inconsistencies arise from multiple factors. Assessment tools variation in sensitivity, and cultural attitude differences toward mental health, and BC across nations affect prevalence and reporting. Sample characteristics, such as our age distribution, marital status, non-metastatic patients versus Guo et al.’s metastatic patients, and unexplored factors like other comorbidities or cancer progression may also influence outcomes.

### Implications

In clinics, the 46.6% depression rate, 56.9% anxiety rate, and 51.9% stress rate signal a clear need to check patients’ mental health regularly. Nurses and oncologists can use the DASS-21 questionnaire during routine follow-ups, as it is quick and has already been proven effective in our study, with a good Cronbach’s alpha reliability. If patients score high, they can be referred to a counselor or social worker already on the hospital team. For rural patients or those who cannot travel, phone check-ins can serve as a substitute for in-person visits, using the same DASS-21 questions to identify issues. This helps keep costs down while identifying problems early.

For public health, our data shows urban residents have higher odds of depression and stress, as do those with inadequate perceived income and mastectomy patients. Local health offices can set up free peer support groups in cities, targeting these groups. For example, a weekly meet-up at a community center can let mastectomy patients share tips on coping with body image changes. Pamphlets with stress management advice, such as breathing exercises, can be distributed at clinics or mailed to low-income patients, leveraging existing mail systems. These steps utilize resources already available, thereby avoiding significant expenses.

Additionally, in clinical and population-based settings, non-pharmacological interventions offer practical, cost-effective ways to manage depression, stress, and anxiety in BC patients. Based on evidence from multiple studies, several strategies stand out for their accessibility and impact. In clinics, physical activity is a straightforward option. A meta-analysis of 26 studies with 2105 participants showed improved quality of life (Hedges’ g = 0.67) and reduced anxiety (Hedges’ g = -0.28) in BC survivors. Clinicians can recommend that patients walk for 30 min most days or join a low-cost yoga class, which requires no special equipment and can be done at home or in a group^40^. Music therapy also proves effective, with 13 studies involving 1326 patients showing reductions in anxiety (SMD = -0.82) and depression (SMD = -0.76)^41^. Patients can listen to pre-recorded tracks for 20–30 min daily, using a smartphone or inexpensive CD player, guided by staff to choose calming selections.

Mindfulness-based interventions (MBIs), evaluated in 19 studies involving 2139 participants, were found to lower depression (*g* = 0.48)^42^. Nurses can teach simple breathing exercises during appointments, encouraging patients to practice them for 10 min daily at home. Art therapy, assessed in 9 studies with 754 patients, reduced anxiety and depression (SMD = -0.48)^43^. Clinics can provide paper and pencils for patients to draw during wait times or sessions. Cognitive behavioral therapy (CBT), reviewed in 16 studies, consistently decreased depression and anxiety scores (*p* < 0.05 in 14 studies)^44^. Staff can deliver brief CBT techniques in one-on-one talks or use free online tools to guide patients remotely. To make these work, clinics should ask patients what they can realistically do and teach them how to start independently. Public programs can partner with local gyms or charities for space and resources. Tracking progress with quick surveys ensures adjustments if something is not helping.

### Future research directions

Future research can delve deeper into these findings by addressing specific, practical questions. A study could track BC patients over a year, using DASS-21 scores every 2–3 months to see when depression peaks. Another study could test if a 10-minute phone counseling session monthly improved mental well-being comparing it to no calls. Researchers should also explore why patients without a family history of BC in second-degree relatives face higher depression and stress odds; is it shock from an unexpected diagnosis? These focused studies can guide clinics and health officials without needing massive funding.

### Limitations

This study has its limitations, including its cross-sectional design, which prevents establishing causality. Additionally, unmeasured confounding variables, such as social support or other comorbidities, could influence the results. The study also did not explore the impact of treatment duration or recurrence on psychological distress. Third, our inclusion criteria requiring literacy and internet/device access may have excluded vulnerable subpopulations (e.g., elderly, low-income, or rural patients with limited digital access). This could underestimate true prevalence in these groups and limit generalizability. Future research should develop and validate telephone-administered versions of instruments like DASS-21 for inclusive sampling. Finally, previous psychiatric history (e.g., pre-existing diagnoses of depression, anxiety disorders, or other mental health conditions) was not assessed in this study. This data was unavailable in the cancer registry and not collected via our questionnaire. Consequently, we cannot determine whether the reported symptoms of depression, anxiety, and stress were solely attributable to the BC diagnosis or its treatment, or if they represent exacerbations of pre-existing conditions. While this is a common limitation in cross-sectional prevalence studies focusing on the cancer population ‘at a glance,’ it restricts causal inferences regarding BC as the sole originator of the psychological distress observed.

## Conclusion

In this study of BC patients in Iran, we observed alarmingly high rates of depression (46.6%), anxiety (56.9%), and stress (51.9%). Several factors significantly increased psychological vulnerability: absence of family history, urban residency, perceived income inadequacy, and mastectomy (versus breast-conserving surgery). These findings highlight the critical need to integrate routine psychological screening using validated tools like DASS-21 into standard oncology care pathways, particularly for identified high-risk subgroups. Beyond screening, our results call for targeted interventions addressing modifiable risk factors, such as financial counseling services, urban support programs countering social isolation, and preoperative psychological preparation for mastectomy candidates.

## Data Availability

The data are available upon reasonable request from the corresponding author.
